# From Physical Activity Intention to Behavior: The Moderation Role of Mental Toughness Among College Students and Wage Earners

**DOI:** 10.3389/fpsyg.2021.584760

**Published:** 2021-05-14

**Authors:** Zhenfeng Cao, Yongtao Yang, Weiwei Ding, Zhijian Huang

**Affiliations:** ^1^Principal’s Office, Hainan Provincial Sports Academy, Hainan, China; ^2^Institute of Sports Training Science, Tianjin University of Sport, Tianjin, China; ^3^School of Physical Education, China University of Geosciences, Wuhan, China; ^4^School of Physical Education, Hubei University, Wuhan, China

**Keywords:** theory of planned behavior, physical activity, mental toughness, college students, wage earners

## Abstract

This study explored the correlation between mental toughness (MT) and physical activity (PA), and the moderation role between PA intention and subsequent behavior among college students and wage earners. Five hundred ninety-one college students (251 male, 340 female) aged from 19 to 24 and 285 (157 male, 127 female) wage earners aged from 27 to 58 recruited from seven colleges and five cities in China. A Theory of Planned Behavior (TPB) questionnaire, MT Inventory, and the International PA Questionnaire was completed online. Results showed that attitudes, subjective norms, and perceived behavioral control explained 46.5 and 38.3% variance in PA intention among college students and wage earners separately. Intention predicted PA behavior significantly among college students and wage earners. Structural equation modeling indicated that the TPB model and the moderation model have an adequate to good fit except the TPB model among wage earners. MT was positively correlated with PA among college students and wage earners and had a significant moderation role in intention-behavior gap among college students and partially affected the transfer of PA intention to behavior among college students. Individuals with high MT had high levels of PA regardless of intention, while PA of those with low MT was low and unstable. Future research should further explore the correlation between intention and PA and the moderation role of MT in different populations using a longitudinal study in order to better understand the correlation between intention and PA, and the transition from intention to PA and better guidance PA intervention to promote PA.

## Introduction

Physical activity (PA) refers to any type of PA with energy consumption due to skeletal muscle contraction, such as daily work, housework, physical exercise, and activities for entertainment (Caspersen et al., 1985). The health benefits of PA have been the focus of researchers across the world for many decades. Studies have found that PA is beneficial for physiological and mental health among different populations in western countries (Hallal et al., 2006; Biddle and Asare, 2011; Biddle et al., 2019; Luan et al., 2019) as well as in China (Sun et al., 2014; Yang et al., 2015, 2021). However, many people still rarely participate in PA in their daily life (World Health Organisation, 2014). Globally, about 27.5% of adults and 81% of teenagers do not reach the level of PA recommended by the WHO.

In China, according to the 2019 General Administration of Sport survey, only 33.9% of adults participate in PA (General Administration of Sport of China, 2018) in China. Another two studies found that only 33.6% of urban children and adolescents participate in PA (Jia et al., 2012), and there was low level of PA in Chinese residents, especially in obese people (Tian et al., 2016). According to the National Health and Family Planning survey in 2019, PA in Chinese adults has decreased by nearly half in the past 30 years. Therefore, given its extensive physical and psychological benefits, it is essential to promote public health through regular PA. In the past few decades, research explained PA behavior by looking at individual internal factors, environmental factors, and the characteristics of the PA itself. This led to the development of models, such as the Health Belief Model (Rosenstock, 1974), Theory of Planned Behavior (TPB; Ajzen, 1991), Trans-theoretical Model (Prochaska and Diclemente, 1983), or the integration of several theoretical models to explain PA.

TPB is important in the explanation of health-related behaviors such as PA behavior, which considers that “intention” is the primary determinant of health-related behaviors (Ajzen, 1991). Originated from the Theory of Reasoned Action, TPB suggests that behavioral intention is determined by one’s subjective norms and attitudes toward the behavior. Perceived behavioral control is added to the TPB to better explain behavior intention (Ajzen, 1985), with attitudes, subjective norms (SN), and perceived behavioral control (PBC) as the three major contributing factors.

• Attitudes reflect an individual’s cognition and evaluation of the behavior, which could be positive or negative.
• SN emanates from influential individuals, especially family members, leading to social pressure.
• PBC is a concept similar to self-efficacy, which refers to an individual’s belief in his or her own behavioral ability.

Many international studies (Ajzen, 2001; Armitage, 2005; Rhodes and Bruijn, 2013; Maccann et al., 2015; Stolte et al., 2017; Cheng et al., 2019), as well as studies in China (Xu et al., 2018; Wang and Zheng, 2020; Zhu et al., 2020) found that TPB variables could explain the variation of behavioral intention, with attitudes and PBC making a greater contribution than SN to the PA intention of an individual (Li, 1999; Feng and Mao, 2014; Gucciardi, 2015; Hannan et al., 2015; Wang and Zheng, 2020). Some studies also used TPB to promote PA (Valerie et al., 2019; Marashi et al., 2020; Sanaeinasab et al., 2020). A meta-analysis also established that attitudes and PBC had a moderate predictive effect on PA intention but that SN were limited (Hagger et al., 2002). Although TPB believed that intention was the cause of subsequent behavior, some researchers found that intention only explains 22% to 27% of the variation of behavior in real-life situations (Ajzen, 2001; Hagger et al., 2002; Mceachan et al., 2011); other studies even found weak or no correlation between intention and subsequent behavior (Plotnikoff et al., 2012; Maher et al., 2017). A meta-analysis also found that only 54% of the “intenders” successfully performed their intended behavior, and those in the samples who failed to translate intention into PA (36%) were nearly twice the number of those with no intention of being active (21%) (Rhodes and Bruijn, 2013). The result suggested that many individuals had a positive intention, but they rarely participated in PA. This is opposite to the traditional view that intention is the primary cause for behavior (Fishbein and Ajzen, 2005). It appears that although the intention may be necessary, alone, it is not sufficient to influence behavior (Rhodes and Bruijn, 2013), especially given the dynamic nature of PA intention (Conroy et al., 2011).

As a result, researchers began to focus on variables which moderated the PA intention-behavior gap, e.g., self-efficacy, extraversion, habit (Rhodes and Dickau, 2012; Rhodes and Yao, 2015), self-regulation, automaticity (Rhodes and Bruijn, 2013; Juliane et al., 2016), self-determined motivation (Feng and Mao, 2014; Robbins et al., 2019; Hernández et al., 2020), planning (Juliane et al., 2016; Xu et al., 2018), emotion or affective judgments (Mohiyeddini et al., 2009; Rhodes and Yao, 2015; Maher et al., 2017; Xu et al., 2018), trait self-control (Pfeffer and Strobach, 2017; Pfeffer et al., 2019), action control (Sniehotta et al., 2005; Zhang and Mao, 2016), and mental toughness (MT) (Gucciardi, 2015; Hannan et al., 2015).

Mental toughness refers to an individual’s capacity to cope with difficult life circumstances (Clough et al., 2002) and is conceptualized as “a personal capacity to produce consistently high levels of subjective (e.g., goal progress) or objective performance (e.g., sales and race time) despite daily challenges and stressors as well as overwhelming adversities” (Gucciardi et al., 2015). It is an important factor that may influence PA and the transition of intention to behavior, owing to its positive trait-like or state-like characteristics. MT can give individuals the perception of strength and improve their behavioral motivation level (Crust et al., 2014; Gucciardi et al., 2015) and also influence the way they evaluate stress, challenges, and adversities in specific situations (Gucciardi et al., 2009), which ensures that they are better focused on the implementation of the plan and goals (Gucciardi, 2010). Moreover, individuals with high MT believe in their ability to participate in PA and have the determination and commitment to actively pursue and achieve PA goals (Crust et al., 2014; Gucciardi et al., 2015). Thus, researchers suggest that “MT is likely to guide the formation of behavioral intention, and also influenced the likelihood of an individual enacting those behaviors” (Crust et al., 2014; Gucciardi, 2015). Previous studies have found a positive correlation between MT and PA (Crust et al., 2014; Gucciardi, 2015; Brand et al., 2016; Serge et al., 2016; Eskandarnejad, 2018; Vaughan et al., 2018; Hegerstad et al., 2019; Cooper et al., 2020; Roncone et al., 2020).

Given that many individuals with PA intentions are still inactive, a high level of PA among individuals may be due to the smaller intention-behavior gap being reduced by high MT. Two studies explored the moderation role of MT in the intention-behavior gap (Gucciardi, 2015; Hannan et al., 2015). One study found that PA intention appeared to have a more substantial effect on PA among individuals with high MT than those with low MT (Gucciardi, 2015), while another study found a non-significant moderation effect of MT, but PA intention is also positively correlated with PA in moderate- and high-mental-toughness individuals (Hannan et al., 2015). These two studies found that moderate and high levels of MT promote the transition of PA intention to behavior. However, participants in the study by Hannan et al. (2015) were community participants and undergraduate students aged from 17 to 63 years, and in Gucciardi (2015), they were individuals aged between 18 and 69 years with knee pains. This study considers that age and social identity may be related to PA intention and behavior.

Physical activity intention and behavior may vary according to the differences in age and social identity. In a recent study, there was no significant difference in the MT of students with high PA compared with those already in the workforce (wage earners), and the positive correlation between MT and PA was only found in students, not in wage earners (Eskandarnejad, 2018). Compared with wage earners, college students have more time and convenient exercise facilities and have a high PA level. In comparison, wage earners often have less time and more working pressure and family commitments, which influence their participation in PA. Moreover, MT generally increased with age (Marchant et al., 2009; Lin et al., 2017). Therefore, we consider that the contribution of TPB constructs on intention and the correlation of MT, PA intention, and behavior may be different among college students and wage earners.

The purpose of this study was to explore the correlation of TPB constructs, MT, and PA behavior among college students and wage earners in China. The study first analyzed the correlation of attitudes, PBC, SN, PA intention, and behavior and hypothesized that attitudes, PBC, and SN can significantly predict PA intention, and intention could also predict PA. Second, we analyzed the moderation role of MT in the PA intention-behavior gap among the two separate groups. We hypothesized that MT correlated with PA in both college students and wage earners and was a critical moderator which influenced the transition of PA intention to behavior.

## Materials and Methods

### Participants

Ethical approval was first obtained from the Human Research Committee of Tianjin University of Sport. A sample of 591 college students (251 male, 340 female) aged from 19 to 24 and 285 wage earners (male 158, female 127) aged from 27 to 58 was recruited from seven colleges and five cities in China based on a convenience sample principle. Students from physical education and sports training were excluded from this study.

### Procedure

The study adopted a prospective design with data being collected in two steps at a 7-day interval. An online version of all questionnaires was designed by the first author. All participants volunteered for this study and joined a WeChat group in order to collect the data of PA. All participants were asked to undertake informed instruction before data collection and then complete the questionnaires.

First, participants finished the questionnaires online, including demographic information, such as age, sex, height, and weight, TPB questionnaire, and the MT Inventory. One week later, all participants received a message that asked them to scan the WeChat QR code to complete the International PA Questionnaire. Twelve students and nine wage earners were excluded from further analysis due to lack of data.

#### Constructs of the Theory of Planned Behavior

Participants completed the Chinese version of TPB questionnaires including four constructs: intention, attitudes, subject norms, and PBC (Hu, 2008). Intention reflects the will a person plans to exert to perform PA and evaluate by using three 6-point Likert items from 1 (strongly agree) to 6 (strongly disagree) (e.g., “I plan to do at least three physical activities a week for more than 20 min each time in the next 4 weeks”), attitudes reflect a person’s affective evaluations of the behavior and evaluate by using five 6-point Likert items (enjoyable–non-enjoyable; pleasant–unpleasant; comfortable–uncomfortable; useful–un-useful; important–unimportant) (e.g., “in the next 4 weeks, at least three times a week for more than 20 min of PA are……), SN reflect perceived social pressure a person faces to engage in PA and evaluate by using three items (e.g., “most people who are important to me want me to participate at least three times PA a week for more than 20 min at a time”), and perceived behavior control (PBC) reflects one’s belief in his ability to perform the PA and evaluate by using three items (e.g., “can you accept at least three times PA a week for more than 20 min in the next 4 weeks”). The internal consistency coefficients of the four dimensions are 0.813, 0.797, 0.893, and 0.817, respectively.

#### Mental Toughness

The Mental Toughness Inventory (MTI) (Gucciardi et al., 2015) is a self-report measure containing eight items to evaluate a person’s tendency to cope with the demands of stressors. Participants’ responses are evaluated on a seven-point Likert scale from 1 (False, 100% of the time) to 7 (True, 100% of the time). A total score is calculated by summing the eight items, with the higher the total score, the stronger the MT. The Chinese version of MTI has a good structural validity (Gucciardi et al., 2016; Li et al., 2019). In order to make the items of the MT closer to the situation of PA, some items were changed accordingly. For example, “I believe I have the ability to achieve my goals” was modified to “I believe I have the ability to achieve PA or exercise goals,” “I believe I have the ability to achieve goals” was modified to “I believe I have the ability to achieve PA or exercise goals,” and “When I perform a task, I can control my focus of attention” was modified to “I can control my focus of attention when participated in PA.” Results showed that the revised MTI still had a good structural validity, χ^2^/df = 1.84, CFI = 0.998, TLI = 0.995, RMSEA = 0.046, SRMR = 0.005. The internal consistency coefficient of the MTI is 0.981.

#### International Physical Activity Questionnaire

The brief edition of the International Physical Activity Questionnaire (IPAQ-Chinese) is a self-report questionnaire used to measure walking, moderate PA, and vigorous PA at work and home and for leisure in 7 days. The volume of total PA is computed in METs—min/week—and converted to MET minutes through weighting each kind of activity by its energy requirements by using formula “physical activity = 3.3 × cumulative low intensity activity time per week + 4.0 × cumulative medium intensity activity time per week + 8.0 × cumulative high intensity activity time per week.” The PA questionnaire has been internationally validated as a reliable assessment of PA for adults aged 18–65 (Craig et al., 2003).

### Data Analyses

All variables were presented as mean and standard deviation. Pearson analysis was used to analyze the correlation of attitudes, PBC, SN, PA intention, and behavior. A confirmatory factor analysis was conducted using Mplus 6.0 to test the factorial validity of the MT questionnaire. We also conducted structural equation modeling using Amos 22.0 to examine whether the model from the TPB constructs to PA fit the data, based on the standard for assessing adequate model fit. CFI and TLI > 0.90, RMSEA and SRMR < 0.08 (Browne and Cudeck, 1992). More stringent cutoff values (CFI and TLI > 0.95, RMSEA and SRMR < 0.06) represented a good model fit (Hu and Bentler, 1999). Simultaneous multiple regression analysis was carried out to investigate whether attitudes, SN, and PBC could predict PA intention using IBM SPSS Statistics 20.0. Moderated regression analysis, using PROCESS 3.0 (Hayes, 2012), analyzed whether MT moderated the relationship between PA intention and behavior. Covariates such as age, gender, and BMI were analyzed in the multiple regression process.

## Results

### Descriptive Statistics

Table 1 presents the mean and standard deviation of attitudes, SN, PBC, intention, MT, and PA. Participants reported positive attitudes to PA; high pressure led to participation in PA (SN), high beliefs in their ability (PBC) of participating in PA, and strong intention toward PA. Based on classification guidelines, 35.6% of the college students’ physical activities were categorized as high, 54.6% as moderate, and 9.8% as low. Of the wage earners, 20.3% were categorized as high, 66.3% as moderate, and 13.4% as low.

**TABLE 1:** Mean and standard deviation for attitudes, subjective norms (SN), perceived behavioral control (PBC), intention, mental toughness (MT), and physical activity (PA).

		**College students**		**Wage earners**	
		**(*N* = 579)**		**(*N* = 276)**	
**Theory of Planned Behavior**					
	Intention	13.66	3.46	13.70	2.93
	SN	13.74	3.28	15.58	3.02
	PBC	13.99	3.80	14.78	3.73
	Attitudes	24.20	5.93	26.05	4.79
MT		38.48	14.12	42.55	13.03
PA		3187.86	2887.55	2689.39	2233.46

### The Correlation Between TPB Variables and Intention

Table 2 shows a positive relationship between attitudes, SN, PBC, and intention among college students and wage earners. Attitudes and intention had the highest correlation, followed by PBC and SN among college students. Likewise, attitudes and intention also had the highest correlation among wage earners, followed by SN and PBC.

**TABLE 2:** Correlations between attitudes, subjective norms (SN), perceived behavioral control (PBC), and physical activity intention.

	**College students (*N* = 579)**				**Wage earners (*N* = 276)**			
	**01**	**02**	**03**	**04**	**01**	**02**	**03**	**04**
01 Intention	–				–			
02 SN	0.405^**^	–			0.523^**^	–		
03 PBC	0.596^**^	0.613^**^	–		0.492^**^	0.546^**^	–	
04 Attitudes	0.622^**^	0.660^**^	0.855^**^	–	0.568^***^	0.701^**^	0.741^**^	–

*^∗∗^*p* < 0.01.*

Simultaneous multiple regression control of age, gender, and BMI showed that the TPB predictors explained 46.5% variance in PA intention among college students [*F*(6,572) = 84.703, *p* < 0.001]. Attitudes (B = 0.255, β = 0.437, *t* = 6.643, *p* < 0.001, 95% CI [0.179, 0.330]) was the strongest predictor of intention, followed by PBC (B = 0.220, β = 0.241, *t* = 3.860, *p* < 0.001, 95% CI [0.108, 0.331]), while SN (B = −0.030, β = −0.028, *t* = −0.658, *p* < 0.01, 95% CI [−0.119, 0.059]) was not a significant predictor.

The TPB predictors also explained 38.3% of the variance in PA intention among wage earners [*F*(6,269) = 29.427, *p* < 0.001], and the contribution of attitudes, SN, and PBC was different to that of college students. Attitudes (*B* = 0.172, β = 0.281, *t* = 3.365, *p* < 0.01, 95% CI [0.071, 0.273]) and SN (*B* = 0.243, β = 0.250, *t* = 3.746, *p* < 0.001, 95% CI [0.115, 0.370]) were strong predictors of intention, while PBC (*B* = 0.100, β = 0.127, *t* = 0.966, *p* > 0.05, 95% CI [−0.010, 0.209]) was not a significant predictor among wage earners.

### Structural Equation Modeling From the TPB Constructs and the Moderation Model to PA

**FIGURE 1:**
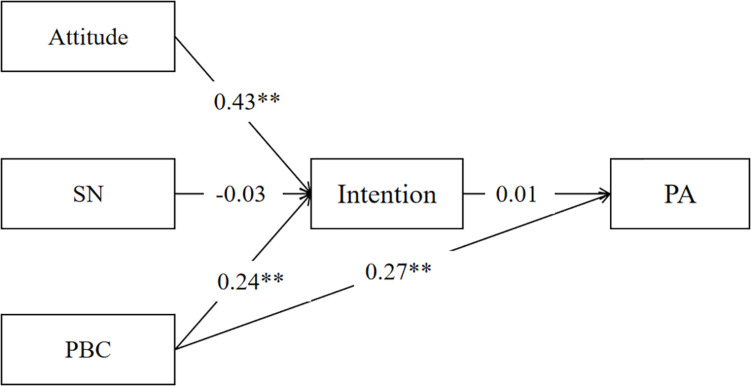
Structural equation model from the TPB constructs to PA among college students. SN, subjective norm; PBC, perceived behavioral control; PA, physical activity; ^*^p < 0.05, ^**^p < 0.01.

**FIGURE 2:**
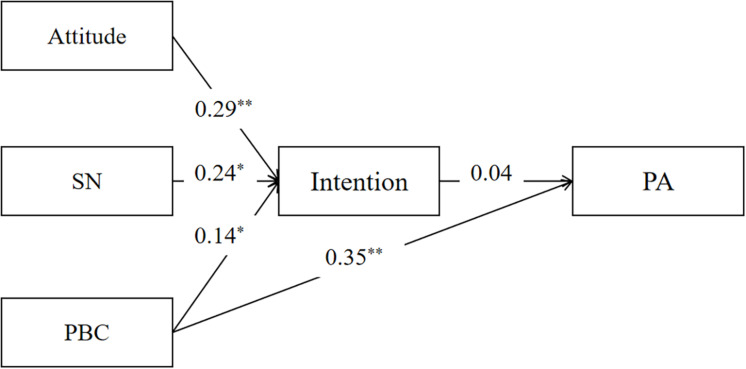
Structural equation model from the TPB constructs to PA among wage earners. SN, subjective norm; PBC, perceived behavioral control; MT, mental toughness; PA, physical activity; ^*^p < 0.05, ^**^p < 0.01.

**FIGURE 3:**
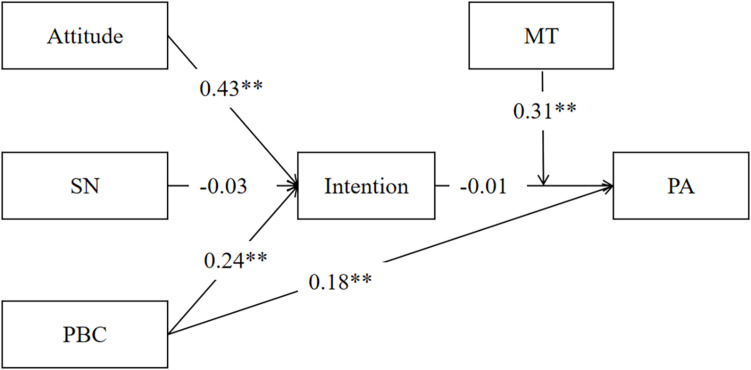
Structural equation model of the moderation role of mental toughness on intention-behavior gap among college students. SN, subjective norm; PBC, perceived behavioral control; MT, mental toughness; PA, physical activity; ^*^p < 0.05, ^**^p < 0.01.

**FIGURE 4:**
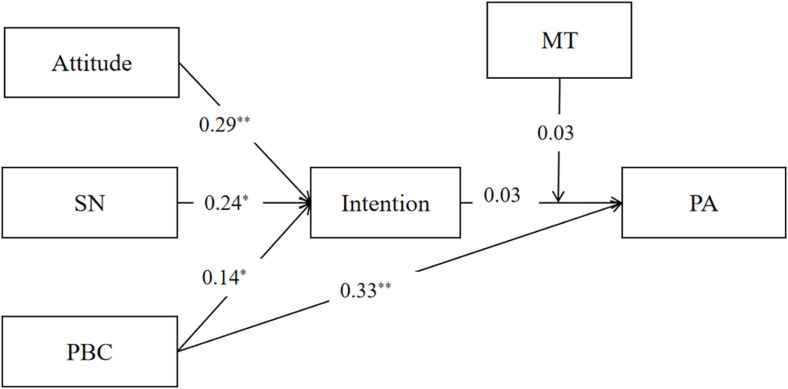
Structural equation model of the moderation role of mental toughness on intention-behavior gap among wage earners. SN, subjective norm; PBC, perceived behavioral control; PA, physical activity; ^*^p < 0.05, ^**^p < 0.01.

Confirmatory factor analysis showed that the TPB constructs to the PA model had an adequate fit across college students (χ^2^/df = 6.850, CFI = 0.992, TLI = 0.959, RMSEA = 0.011). However, the model tested among wage earners did not provide a good fit (χ^2^/df = 0.189, CFI = 1.000, TLI = 1.014, RMSEA = 0.001). Moreover, the moderation model of MT on the intention-behavior gap had a good fit among both college students (χ^2^/df = 1.805, CFI = 0.998, TLI = 0.990, RMSEA = 0.037) and wage earners (χ^2^/df = 2.570, CFI = 0.991, TLI = 0.954, RMSEA = 0.076). The moderation role of MT was influential on the intention-behavior gap among college students. See Figures 1–4.

### Intention and Mental Toughness as a Predictor of Physical Activity

Correlations between intention, MT, and PA that were entered into the moderated regression model are presented in Table 3. MT correlated closely with intention among both college students and wage earners. Intention also correlated significantly with PA in the two independent samples. Both MT (*t* = 3.817, *p* < 0.001, 95% CI [55.819, 174.188]) and intention (*t* = 2.523, *p* < 0.05, 95% CI [49.373, 396.505]) made substantial contributions to the PA among college students. Multiple linear regression analysis showed that PA intention could clearly explain 7.4% of the variance of PA among college students, [*F*(4,574) = 12.491, *p* < 0.001. *t* = 3.696, *p* < 0.001], 95% CI [61.563, 201.190]), and 9.6% of the variance of PA among wage earners [*F*(4,271) = 8.321, *p* < 0.001. *t* = 3.058, *p* < 0.01], 95% CI [49.198, 227.026]). In addition, the moderation model explained a substantial portion of variance (16.37%) of PA among college students [*F*(6,572) = 18.655, *p* < 0.001] and 14.09% variance of PA among wage earners, [*F*(6,269) = 7.335, *p* < 0.001]. It appeared that MT made little contribution to PA among wage earners (*t* = 1.920, *p* = 0.056, 95% CI [−2.295, 183.714]).

**TABLE 3:** Correlations between intention, mental toughness (MT) and physical activity (PA).

	**College students (*N* = 579)**			**Wage earners (*N* = 276)**		
	**01**	**02**	**03**	**01**	**02**	**03**
01 Intention	–			–		
02 MT	0.315^**^	–		0.354^*^	–	
03 PA	0.166^**^	0.335^**^	–	0.216^**^	0.249^**^	–

*^∗∗^*p* < 0.01.*

**FIGURE 5:**
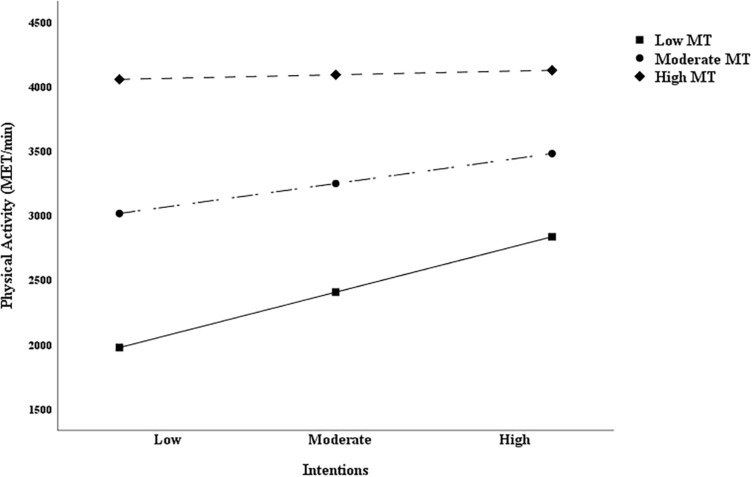
Simple slopes for physical activity intentions and behavior relations at different mental toughness levels among college students.

**FIGURE 6:**
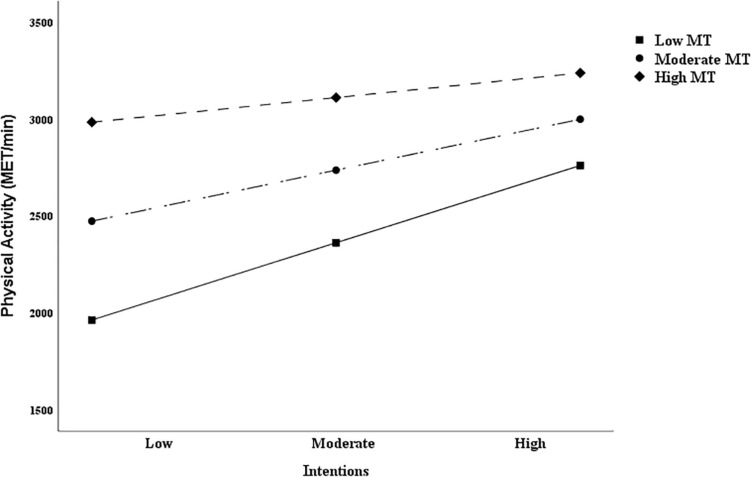
Simple slopes for physical activity intentions and behavior relations at different mental toughness level among wage earners.

Based on the suggestion proposed by Robinson et al. (2013), “researchers investigating moderator variables should begin with an examination of the simple slopes rather than relying on a significant interaction term.” For this reason, this study also used simple-slope analysis to further explore the moderating effect of MT in the intention-behavior gap. Results suggested that the correlation between intention and PA was significant at low levels of MT (*t* = 2.684, *p* < 0.01, 95% CI [33.366, 215.427]), but not significant at moderate levels (*t* = 1.917, *p* = 0.056, 95% CI [−1.672, 136.315]) or high (*t* = 0.226, *p* > 0.05, 95% CI [−78.796, 99.244]) of MT among college students (Figure 5), and also the similar results among wage earners (Figure 6), with low levels of MT (*t* = 2.159, *p* < 0.05, 95% CI [12.021, 260.436]), moderate (*t* = 1.794, *p* > 0.05, 95% CI [−8.771, 188.344]), and high (*t* = 0.531, *p* > 0.05, 95% CI [−117.246, 203.935]).

## Discussion

Based on the TPB, *intention* is the primary determinant for health-related behavior (Ajzen, 2001). However, the correlation between PA intention and behavior is often mediated in natural situations by factors such as MT, self-efficacy, emotions, and action plans. Researchers consider that MT plays an important role in promoting PA behavior. It may be an important factor that affects PA intention and can be seen in its transition to behavior in different populations. This study explored the correlation between TPB constructs and PA, and the moderating role of MT in PA the intention-behavior gap among college students and wage earners.

The TPB constructs could be relevant in explaining the variance of PA intentions, with 46.5% among college students and 38.3% among wage earners, respectively, which was consistent with previous studies (Armitage, 2005; Conner et al., 2011; Rhodes and Bruijn, 2013; Gucciardi, 2015; Hannan et al., 2015; Stolte et al., 2017; Xu et al., 2018; Wang and Zheng, 2020; Zhu et al., 2020). This study also found the different contribution of TPB constructs on intention among college students and wage earners. That is, attitudes and PBC could be crucial in the prediction of PA intention among college students, with attitudes having the greatest power, followed by PBC.

PBC could significantly predict behavioral intention among wage earners. This was partially consistent with previous results (Hagger et al., 2007; Gucciardi, 2015; Hannan et al., 2015). Studies had found that attitudes and PBC were the main predictors of PA in males, while SN was the main predictor of PA in females (Hasnan et al., 2014). The predictive effect of PBC and SN was proportional to age, and the predictive effect of attitudes was inversely proportional (Waekel et al., 1994). We speculated that PA intention was influenced by the identity of individuals in their daily life. Compared with college students, wage earners live in a more socially complicated environment and face with greater pressure in daily life. Social support from others, especially family members, is a more important factor influencing their PA intention than that of college students. This study showed that the contribution of SN on intention among wage earners was greater than that of college students, but attitudes among wage earners can rarely be transformed into PA without sufficient social support. Studies also found that expectation of a negative emotional experience was a significant predictor of PA intention, and its impact exceeded the TPB constructs (Wang, 2011; Rhodes and Yao, 2015; Maher et al., 2017). The effect of self-efficacy on intention was also greater than that of the TPB constructs (Rhodes and Dickau, 2012; Rhodes and Yao, 2015; Wang and Zhang, 2016), which was similar with one meta-analysis that anticipated that regret is the strongest contributor to intention (Sandberg and Conner, 2008) after controlling the TPB constructs. Therefore, TPB constructs are not enough to accurately explain individuals’ PA intention; research should further extend the theoretical model of TPB so as to better understand individuals’ behavior intention.

Mental toughness positively correlated with PA both among college students and wage earners. The results further supported the view that MT was a determinant of PA (Gerber et al., 2012, 2015; Gucciardi, 2015; Serge et al., 2016). Previous studies found that people with high MT showed higher levels of commitment, motivation, resilience, and self-adjustment (Crust et al., 2014). These characteristics were important mechanisms as to why people with high MT were more likely to participate in PA. In addition, people with high MT tended to have greater determination, believed in their ability to participate, and had more coping resources, such as social support (Gill et al., 2017), which could help to overcome adversities encountered in achieving PA goals.

Excellent executive control and self-monitoring abilities (Hannan et al., 2015; Pfeffer and Strobach, 2017) of high MT individuals were also factors promoting PA, with emotional experience also considered to be a variable. Studies found that negative emotional experience and PA behavior showed a two-way negative relationship (Cushing et al., 2018), and positive emotional experience could predict individual PA behavior in the natural environment (Liao et al., 2017), while negative emotional experiences such as stress and fatigue were associated with reduced PA (Stults-Kolehmainen and Sinha, 2014; Jones et al., 2017). Thus, one of the important reasons for a low level of PA in low MT individuals may be the high level of negative emotions such as stress and fatigue experienced in daily life. In conclusion, most of the current studies found a positive correlation between MT and PA, although some studies found inconsistent results (Hannan et al., 2015; Eskandarnejad, 2018).

Intention can significantly predict PA among college students and wage earners. When MT was added to the intention-behavior gap as a moderation variable, its contribution in predicting PA increased, from 7.4% of the variance to 16.37% among college students, and 9.6% of the variance to 14.09% among wage earners. Previous studies found that MT could give the individual a perception of strength, and improved their behavioral motivation level (Crust et al., 2014; Gucciardi et al., 2015), and especially influenced the way stress, challenges, and adversities were evaluated (Gucciardi et al., 2009), which ensured a better focus on the implementation of the plan and goals (Gucciardi, 2010).

Individuals with high MT believe in their ability to participate in PA and have the determination and commitment to actively pursue and achieve PA goals (Crust et al., 2014; Gucciardi et al., 2015). Therefore, high MT could promote PA, and intention has no direct effect on the two independent samples’ moderation model. Importantly, the moderation role of MT among college students was significant, while the moderation effect among wage earners was not. These results were partially consistent with the hypothesis that the moderation model had its greatest predicting power among college students. The different identities in the two independent samples’ daily lives, such as less time, high working pressure, family affairs, and age differences in MT mentioned above may be the crucial reasons. Figure 1 also shows that the direct contribution of PBC on PA decreases with MT added to the model, which indirectly reflected the importance of the contribution of MT on PA.

Further analysis using the simple slopes showed that PA of low-mental-toughness individuals was more susceptible to PA intention, while PA of both college students and wage earners with high MT was scarcely influenced by intention, suggesting that individuals with high MT undertook high levels of PA regardless of intention. The result was inconsistent with previous studies. Gucciardi (2015) found that the PA intention-behavior gap became the smallest when MT was high, and PA in low MT was less affected by intention. Hannan et al. (2015) also found that intention was significantly and positively correlated with PA when MT was moderate or high, but this study did not find any compelling evidence for the contribution of MT on PA. Three reasons could explain the differences of the results of above three studies.

First, cultural differences may be a possible consideration. Individuals in Western countries may have a more positive PA habit and often participate in PA (Zhang and Tang, 2019), which could improve MT. For example, the PA level reported in Hannan et al. (2015) was high (5288.64 ± 3936.14), while in this study PA level was lower (college students: 3187.86 ± 2887.55, wage earners: 2689.39 ± 2233.46). Moreover, the TPB was perhaps not universal in explaining PA because its constructs were the cultural values that tend to be endorsed by different populations in different cultures (Hagger et al., 2007).

Second are differences in the characteristics of the samples. The samples in Hannan et al. (2015) were community participants and undergraduate students aged from 17 to 63 years, which may ignore the effects of identity differences, while participants in Gucciardi (2015) were individuals with knee pain aged from 18 to 69 years. Therefore, age, identity and role characteristics, and health conditions should be considered to explain the results in the differences in MT and the variables of TPB in different samples. A previous study had found that the relationship between MT and PA varies according to population characteristics (Eskandarnejad, 2018). This study also revealed the differences of TPB variables on intention and PA between college students and wage earners, which further supported this view.

Third, the difference of the TPB questionnaire applied in Hannan et al. (2015) used a TPB questionnaire with a seven-point Likert rating scale on 20 items, Gucciardi (2015) used a TPB questionnaire with a six-point Likert scale on 13 items, and our study used the Chinese version TPB questionnaire with a six-point Likert scale on 14 items. Differences in the scale items, especially in scoring criteria, may be another reason for the difference of results in different studies. It should be noted that this study had a limitation in that the measurement time did not match the 4-week timeframe set by TPB theory. A 7-day interval, not a month (4 weeks) interval, of data collection between intention and PA may influence the accuracy of PA data. Although previous studies also used a 7- (Hannan et al., 2015) or 14-day (Gucciardi, 2015) interval between data collections, the item was modified as “I will attempt to do the recommended minimum amount of PA over the next week.” Therefore, it is important to consider the measurement interval between PA intention and behavior. If the PA measurement time does not allow a month interval, researchers should consider matching the measurement time between intention and subsequent behavior.

## Conclusion

In conclusion, TPB constructs could be significant in the prediction of PA intention both among college students and wage earners. MT is an important and positive predictor of PA, which suggests that researchers should consider the role of MT in intervention in the PA of adolescents. MT intervention may be an effective strategy to promote PA. While intention directly affects PA, its contribution to behavior decreases once MT is added to the mode: MT is an important variable that influences the transition of intention to PA. Individuals with high MT display high levels of PA regardless of intention, while the PA of those with low MT remains low and unstable. Future research should further explore the correlation between intention and PA and the moderation role of MT among different populations using a longitudinal study to better understand the intention-behavior gap.

## Data Availability Statement

The raw data supporting the conclusions of this article will be made available by the authors, without undue reservation.

## Ethics Statement

The studies involving human participants were reviewed and approved by the Human Research Committee of Tianjin University of Sport. The ethics committee waived the requirement of written informed consent for participation.

## Author Contributions

YY and ZC contributed equally to this work. Both of them designed this study, oversaw data collection and data analysis. YY and ZH contributed to data interpretation and the drafting and revision of the manuscript. WD was also responsible for data collection. All authors contributed to the article and approved the submitted version.

## Conflict of Interest

The authors declare that the research was conducted in the absence of any commercial or financial relationships that could be construed as a potential conflict of interest.
